# The role of parliamentarians in promoting self-care interventions for sexual and reproductive health and rights: applying COVID-19 lens in the Eastern Mediterranean region

**DOI:** 10.1186/s12961-021-00689-y

**Published:** 2021-04-21

**Authors:** Hala Abou-Taleb, Nada Mohamed, Karima Gholbzouri, Elisa Scolaro, Inaya Ezzeddine, Souhail Alouini, Heba Hagrass, Magdy Morshed, Neil Datta

**Affiliations:** 1 Health Systems Governance, World Health Organization Regional Office for the Eastern Mediterranean, Extension of Abdel Razak El Sanhouri Street, P.O. Box 7608, Nasr City, Cairo, Egypt; 2 Reproductive and Maternal Health, World Health Organization Regional Office for the Eastern Mediterranean, Extension of Abdel Razak El Sanhouri Street, P.O. Box 7608, Nasr City, Cairo, Egypt; 3 Sexual and Reproductive Health and Research, World Health Organization, Avenue Appia 20, 1211 Geneva, Switzerland; 4 National Assembly of Lebanon, Nijmeh Square, Beirut, Lebanon; 5 Assembly of People’s Representatives in Tunisia, Le Bardo, Tunis, Tunisia; 6 House of Representatives of Egypt, 1 Majlis Al-Shaab Street, Kasr El-Aini, Cairo, Egypt; 7 European Parliamentary Forum for Sexual and Reproductive Rights, Rue Montoyer 23, 1000 Brussels, Belgium

**Keywords:** Parliamentarians, Self-care interventions, Sexual and reproductive health and rights (SRHR), COVID-19 pandemic, Disruption of healthcare services, Eastern Mediterranean region

## Abstract

Innovative people-centered care modalities including self-care interventions offer an opportunity to ensure continuity of healthcare services during COVID-19 and in post-COVID-19, as well as contribute to the achievement of universal health coverage. Parliamentarians are uniquely positioned to promote self-care interventions for sexual and reproductive health and rights through their legislative, budget allocation, oversight, and advocacy roles. However, existing health systems governance challenges in the Eastern Mediterranean region such as weak institutions setups, fragmentation of health programs, and limitation of resources could impede parliamentarians’ progress. To address these challenges, the following recommended actions should be considered: (1) promote the adaptation of sexual and reproductive health and rights service packages at primary healthcare level to integrate self-care interventions (2) govern innovative people-centered care channels including self-care interventions; and (3) engage in a dialogue with civil society and communities to meet needs, raise public awareness and generate demand.

## Introduction

Leadership and political will are essential to promoting people’s right to access quality, affordable and acceptable healthcare services. Parliamentarians play a central role in advancing the health agenda forward, including sexual and reproductive health and rights (SRHR). As lawmakers, parliamentarians design, adopt and oversee the implementation of legislation that promote SRHR and equity in health-care provision. They monitor the work of government to ensure the implementation of SRHR policies, strategies, and plans. In addition, parliamentarians engage with different stakeholders and actors to advocate for their constituents’ right to SRHR.

As the COVID-19 pandemic continues to infect and claim the lives of millions of people and cause harm to countries’ economy and social cohesion, it is clear that the world is facing a health crisis unlike any before. However, the impact of the pandemic goes beyond the suffering caused by the rapid spread of the virus itself. Essential services, including sexual and reproductive healthcare, have either been disrupted or suspended in many countries, jeopardizing the health and wellbeing of countless individuals, more so in countries experiencing emergencies [1].

Self-care interventions are an innovative approach to sustain equitable access to quality, affordable and acceptable healthcare services in the context of the COVID-19 pandemic and beyond, as well as contribute to the achievement of universal health coverage (UHC). The World Health Organization defines self-care as “the ability of individuals, families and communities to promote health, prevent disease, maintain health, and cope with illness and disability with or without the support of a healthcare provider” [2].

This commentary outlines the importance of self-care interventions in ensuring the continuity of healthcare services during COVID-19 and beyond in the Eastern Mediterranean region (EMR), and the crucial role that parliamentarians play in promoting self-care interventions for SRHR, including in humanitarian settings.

## The COVID-19 pandemic and the disruption of non-COVID-19 services in EMR

As the number of confirmed cases of covid-19 surges across EMR, it is clearer than ever that no country is immune. As of the 27th of October 2020, almost 3 million cases and more than 75,000 deaths have been reported in the region [3]. This dramatic increase in cases accompanied by rapid spread of coronavirus are presenting numerous challenges even to the most advanced health systems and disrupting the provision of essential healthcare services [1]. In addition, people’s fear of catching the virus, and the nationwide curfews are holding back many individuals from visiting healthcare facilities [4]. This will negatively impact the health and safety of many vulnerable populations who require access to essential health services, including sexual and reproductive healthcare. A study that applied a mathematical model, projects that the disruption of essential health services due to COVID-19 pandemic in Egypt can negatively impact the access of 500,000 women to facility-based deliveries, and 3 million women to family planning services [5].

Self-care interventions offer individuals an opportunity to address their health needs and make an informed decision about their health in a private settings without having to visit a health facility. It is not a new-fashioned concept and it has been exercised by people for thousands of years. Hand washing, and teeth brushing are examples of self-care practices which individuals carry out every day to stay healthy [6]. Self-care measures can be particularly beneficial as they provide access to quality healthcare services for vulnerable populations including those living with disabilities, and enable healthcare workers to provide services to a larger number of patients, especially in countries facing humanitarian crisis where health systems infrastructure is either impaired or non-existent [7]. In the context of COVID-19 and the consequent discontinuity of care, a range of self-care interventions for SRHR including self-administered injectable contraception, HIV self-testing, and telemedicine for prenatal care can protect people’s health and well-being and improve their sexual and reproductive health [6].

## Parliamentarians’ role in the promotion of self-care interventions in EMR

Parliamentarians in EMR are uniquely positioned to promote self-care interventions for better health outcomes generally, and for SRHR particularly. Taking into account regional diversity and COVID-19 lessons learned, parliamentarians can review and propose legislation to support self-care policies and plans at a country level, use gender-responsive and equity-sensitive budgeting tools to ensure adequate allocation of resources to self-care interventions, engage in policy dialogue with relevant healthcare stakeholders to identify feasible self-care interventions, and advocate the benefits of self-care interventions across the different parliamentary committees and among their constituents.

Parliamentarians in EMR are proactively engaging with WHO Regional Office for the Eastern Mediterranean to advocate for adoption of the 2019 WHO Consolidated Guideline on self-care interventions for SRHR. A virtual session was organized in April 2020 to discuss the rapid introduction and scale-up of self-care interventions for SRHR at country level. The session’s discussion captured the need to: (1) facilitate policy dialogue between national governments and parliamentarians on the implementation of self-care interventions for SRHR; and (2) identify needed advocacy and documentation to support the implementation of self-care interventions at a country level.

However, the existing health systems governance challenges in the Region ranging from weak institutions setups, to fragmentation of health programs, and limitation of resources to ensure coverage and quality people-centered care could impede parliamentarians’ promotion of self-care interventions.

The following are few recommended actions to address these challenges and facilitate parliamentarians roles in promoting the introduction of self-care interventions for SRHR.

- Promote the adaptation of SRHR service packages at primary healthcare level to include self-care interventions
- Regulate innovative people-centered care channels including self-care interventions to ensure quality and protection of privacy and confidentiality based on a set of criteria
- Engage with civil society to address populations’ need, mobilize public support for self-care interventions and generate demand

## Conclusion

Self-care interventions offer an opportunity to ensure continuity of healthcare services during COVID-19 and beyond and advancement towards UHC. Parliamentarians in EMR can play a fundamental role in the promotion of self-care interventions for SRHR through their legislative, budget, oversight and advocacy roles.

## Data Availability

Data sharing is not applicable to this article as no datasets were generated or analyzed during the current study.

## Abbreviations

EMR: Eastern Mediterranean region
SRHR: Sexual and reproductive health and rights
UHC: Universal health coverage
